# Assessment of the iodine nutritional status among Chinese school-aged children

**DOI:** 10.1530/EC-19-0568

**Published:** 2020-04-15

**Authors:** Ning Yao, Chunbei Zhou, Jun Xie, Xinshu Li, Qianru Zhou, Jing Chen, Shuang Zhou

**Affiliations:** 1 Chongqing Center for Disease Control and Prevention, Chongqing, People’s Republic of China

**Keywords:** school aged children, iodine nutrition, iodine deficiency disorders, goiter, cross-sectional research

## Abstract

**Objective:**

The remarkable success of iodine deficiency disorders (IDD) elimination in China has been achieved through a mandatory universal salt iodization (USI) program. The study aims to estimate the relationship between urinary iodine concentration (UIC) and iodine content in edible salt to assess the current iodine nutritional status of school aged children.

**Methods:**

A total of 5565 students from 26 of 39 districts/counties in Chongqing participated in the study, UIC and iodine content in table salt were measured. Thyroid volumes of 3311 students were examined by ultrasound and goiter prevalence was calculated.

**Results:**

The overall median UIC of students was 222 μg/L (IQR: 150-313 μg/L). Median UIC was significantly different among groups with non-iodized salt (iodine content <5 mg/kg), inadequately iodized salt (between 5 and 21 mg/kg), adequately iodized (between 21 and 39 mg/kg) and excessively iodized (>39 mg/kg) salt (*P* < 0.01). The total goiter rate was 1.9% (60/3111) and 6.0% (186/3111) according to Chinese national and WHO reference values, respectively. Thyroid volume and goiter prevalence were not different within the three iodine nutritional status groups (insufficient, adequate and excessive, *P* > 0.05).

**Conclusions:**

The efficient implementation of current USI program is able to reduce the goiter prevalence in Chongqing as a low incidence of goiter in school aged children is observed in this study. The widened UIC range of 100–299 μg/L indicating sufficient iodine intake is considered safe with a slim chance of causing goiter or thyroid dysfunction. Further researches were needed to evaluate the applicability of WHO reference in goiter diagnose in Chongqing or identifying more accurate criteria of normal thyroid volume of local students in the future.

## Introduction

Iodine deficiency disorders (IDD) indicate the broad range of harmful effects due to low level of thyroid hormones in the blood caused by an inadequate dietary supply of iodine (1, 2). Pregnant women and young children are more vulnerable to iodine deficiency because the former require higher iodine intake to maintain both maternal and neonatal normal neurodevelopment, and the latter were more sensitive to even mild iodine deficiency (1). Absorption of iodine at sufficient level is essential for the fetus and mother’s thyroid glands to function healthily (3). The most devastating consequences of IDD for neonate and child include neonatal hypothyroidism, reproductive impairment, retarded mental and physical development (4), and the decreased survival of children (1). In all age groups, iodine deficiency can cause goiter, an enlargement of the thyroid gland, as it adapts to chronic iodine deficiency (5). IDD have long been a significant public health problem in China, with over 700 million people in the 1990s being iodine deficient (6). Chongqing is a municipality directly under the control of central government, which lies in the south-west of China. In 1994, epidemiological studies conducted in Chongqing demonstrated that 41.5% of schoolchildren aged 7–14 years showed visible signs of goiter with a median urinary iodine concentration (UIC) of 53.1 μg/L, and iodine concentration in drinking water was 3.3 μg/L. Most of the investigated household table salts were non-iodized, and then Chongqing was identified as an endemic area of IDD (7).

In order to control and eliminate IDD, China has adopted salt iodization as its principal control strategy in 1993 and had established mandatory universal salt iodization (USI) program in 1995 (6). With high and sustained levels of national monopoly for the production, wholesale and inspection of iodized salt and well-coordinated, high-quality surveillance, the program has achieved tremendous success in China and effective salt iodization has been recognized as the optimum and long-term solution to the problem (8). China had virtually eliminated IDD by 2000, and the 2010 national evaluation concluded that 28 provinces (including Chongqing) had eliminated IDD and three provinces were ‘almost eliminated’ (5). But regional difference of iodine nutritional status of school aged children and pregnant women among provinces still existed. According to China’s 2011 National IDD Surveillance results (5) and the widened range of 100–299 μg/L as optimal of UIC for school aged children (9), in 20 provinces including Chongqing, both pregnant women and school aged children have optimal iodine intakes of China’s 31 provinces. While in seven provinces the median UIC level of school aged children were in the range of 100–299 μg/L but with pregnant women being less than 150 μg/L (six provinces) or ≥250 μg/L (one province). Both iodine deficiency and excess may cause adverse effects in children or during pregnancy.

It is important to launch regular, well-coordinated and high-quality surveillance of iodine status in the most vulnerable group that provides accurate information on the current IDD situation. Goiter prevalence in school aged children and median UIC are currently the two most widely used indicators of IDDs. Urinary iodine excretion is a good marker of current dietary iodine intake, which makes UIC a reliable indicator to assess, monitor and evaluate iodine nutrition for all age groups (1, 4). Thyroid size or goiter is another useful indicator which reflected long-term population iodine nutrition. The present study aims to investigate the current iodine nutritional status of school aged children in Chongqing and to estimate the relationship between UIC and iodine content in edible salt to help determine optimal iodine intake of school aged children.

## Methods

### Study design and sample collection

This cross-sectional study was conducted in 26 of 39 districts/counties of Chongqing from May through October in 2017, which was embedded into the 2016 China’s National IDD Surveillance Program led by National Health Commission of the People’s Republic of China. As the prevalence of goiter of 8–10 years old children in Chongqing in 2016 investigation program was 2.2%, and type I error α and permissible error δ were both set at 0.05, the minimal sample size for the target town was 32 according to the equation for simple random sampling. Design effect (*k*) was set at 1.25 considering that cluster sampling was used instead of simple random sampling at town level for convenience.

Sampling procedures were done according to China’s national surveillance guidelines of IDD program (10). In each county, five towns were randomly selected from five different geological locations (east, west, south, north and center). One primary school was randomly chosen from each town if there was more than one school. Forty (40) healthy students aged 8–10 years (20 boys and 20 girls) from one class in each grade were recruited by cluster sampling. Students in adjacent class could be selected as a supplement if there were less than 20 by each gender. Those who have history of thyroid disease or confirmed using thyroid-related medication were not enrolled in the study. For each student, approximately 10 mL of a random spot midstream urine sample was collected in the morning from 08:30 to 12:00 h. Probably 50–100 g of household table salt was also collected from each student’s home.

Students from 14 districts of 26 were chosen to be conducted thyroid ultrasonic examinations for goiter due to limited financial and time resources. Written informed consent was obtained from the principal of the school and verbal informed consent from parents or legal guardians of participating children were normatively recorded. The study was approved by the Ethical Review Committee of Chongqing Center for Disease Control and Prevention (CDC) and all procedures performed in studies involving human participants were in accordance with the ethical standards of the committee.

### Determination of iodine concentration and thyroid size

Iodine content in table salt was measured using a titration method with sodium thiosulphate (GB/T 13025.7-2012) and was expressed as mg/kg. UIC was determined using arsenic-cerium catalytic spectrophotometry (WS/T 107.1-2016). UIC was expressed as μg/L. All the 14 laboratories testing iodine content in salt and UIC previously participated in IDD laboratory quality control network implemented by the national reference laboratory (NRL), which was run by the Chinese CDC. They got the certification renewed annually through standardized operating procedures and testing the proficiency of laboratory technicians to ensure that all laboratories responsible for testing urinary and salt iodine content could provide reliable data. A proportion of 5% of total urine and salt samples were reexamined in reference laboratory of Chongqing CDC for quality control.

According to the ‘guidance on the monitoring of salt iodization programmes and determination of population iodine status’ proposed by United Nations International Children’s Emergency Fund (UNICEF) (9), iodine nutritional levels under 100 μg/L, between 100 and 299 μg/L, and over 300 μg/L were defined as insufficient, adequate and excessive, respectively. In grading deficiency, the levels between 50 and 99 μg/L, 20 and 49 μg/L and under 20 μg/L were assessed as mild, moderate and severe deficiency, respectively. Household table salt were classified into four groups: non-iodized salt with iodine content <5 mg/kg, inadequately iodized when between 5 and 21 mg/kg, adequately iodized when between 21 and 39 mg/kg, excessively iodized when >39 mg/kg (GB 26878-2011) (11).

Thyroid volume was measured by an echocamera named Mindray-M5 (Mindray, Shenzhen, PRC). Thyroid volume was calculated using the rotation ellipsoid formula: length (cm) × width (cm) × thickness (cm) × correction factor (0.479) for each lobe (1, 12). Goiter prevalence was calculated based on both international (WHO) (4, 13) and Chinese standard (14) for thyroid volume in children. For 8-year-old children, thyroid volume measured by ultrasonography exceeding 4.5 mL is defined as goiter, for 9 years and 10 years are separately 5.0 mL and 6.0 mL according to Chinese domestic diagnostic criteria for endemic goiter (WS 276-2007) (14). WHO recommended another reference of the upper limit of normality for thyroid volume defined by a gender-specific 97th percentile (P97) as a function of age and body surface area (BSA). We did not collect weights and heights of the students this time, so thyroid volume adjusted by BSA was not used. The criteria for boys aged 8–10 years, P97 was 3.71, 4.19, 4.73 mL separately, and for girls was 3.76, 4.32, 4.98 mL separately (4). Based on WHO epidemiological criterion for assessing the severity of IDD according to the prevalence of goiter in school aged children (4), a region is identified of non-IDD endemic given the total goiter rate (TGR) in the range of 0–4.9% and is considered for iodine insufficiency if more than 5% of schoolchildren have goiter, further estimated being mild IDD endemic when between 5.0 and 19.9%, moderate when between 20.0 and 29.9%, severe when above 30%.

### Statistical analysis

Data were input in Microsoft Office Excel 2007 and data analysis was performed using SAS 9.13 (SAS Institute Inc.). Kolmogorov–Smirnov (KS) test was used for normality test. Iodine content of table salt, UIC and thyroid volume were expressed as median and interquartile range (IQR). The Wilcoxon test and Kruskal–Wallis test were used to compare UIC and thyroid volume among subgroups. Comparisons of iodine status and goiter rate among subgroups were done using chi-squared test. *P* < 0.05 was considered statistically significant. Spearman correlation analysis was used to determine the relationship between thyroid volume and UIC with iodine content in table salt. The values of thyroid volume, iodine content of table salt and UIC were natural log transformed to assure normality of data and then constructed through multiple linear regression to estimate the relationship between thyroid volume and age, UIC and iodine content of table salt. A curve was formulated with median UICs, thyroid volumes and goiter rates to describe the visualized relationship among them, UIC was grouped as <20, 20–49, 50–99, 100–149, 150–199, 200–299, 300–499, 500–799, 800–999 and ≥1000 μg/L (15) and thyroid volumes and goiter rates of population were calculated for each UIC group.

## Results

### Table salt iodine concentration and UIC distribution

A total of 5565 students aged 8–10 years (2784 boys and 2781 girls) were recruited in this study. All of them provided a casual urine sample. UIC levels and iodine content of household table salt were non-normally distributed by KS test with D statistic of 0.13 and 0.21 respectively (*P* < 0.01). The overall median UIC of students was 222 μg/L (IQR: 150–313 μg/L), indicating the iodine nutritional status of students were adequate. Of all the students, 166 (2.98%) had UIC measurements below 50 μg/L. A total of 5546 table salt samples were collected and detected, the median iodine content of the table salt was 25.5 mg/kg (IQR: 23.1–28.3 mg/kg) with a coverage rate of 99.5% (5520) and a qualified rate of 91.9% (5094) referring iodized table salt. There were 6.5% (361) and 1.2% (65) of iodized table salts specimens detected to be inadequately or excessively iodized, respectively (Table 1).

**Table 1:** UIC according to gender, age and category of table salt.

Characteristics	*n* (%)	Median UIC, μg/L (IQR)	*P*
Gender			<0.01
Boys	2784 (50.0)	236 (160–332)	
Girls	2781 (50.0)	207 (139–294)	
Age			0.53
8 years	1709 (30.7)	219 (149–304)	
9 years	1992 (35.8)	223 (152–319)	
10 years	1864 (33.5)	222 (147–316)	
Table salt			<0.01
Non-iodized^a^	26 (0.5)	144 (87–208)	
Inadequately iodized^b^	361 (6.5)	239 (159–349)	
Adequately iodized	5094 (91.9)	221 (150–312)	
Excessively iodized	65 (1.2)	189 (119–261)	

Wilcoxon test was used to compare UIC between genders. Kruskal–Wallis test was used to compare UIC among age groups and different salt consuming groups. *P* < 0.05 was considered statistically significant.

^a^*P* < 0.001 and *P* = 0.002 compared with inadequately iodized and adequately iodized group; ^b^*P* = 0.0066 and *P* = 0.0016 compared with adequately iodized and excessively iodized group.

IQR, interquartile range; UIC, urinary iodine concentration.

Boys had a significantly higher median UIC than girls (*P* < 0.01), and both were indicating adequate iodine intake. The median UIC was not statistically different among age groups (*P* = 0.53). Students who provided household table salts that were detected to be non-iodized or excessively iodized had lower median UIC than those provided inadequately or adequately iodized table salts. Multiple comparisons showed there was no statistical difference between UIC of students who consumed excessively iodized salt with those of non-iodized or adequately iodized salt (Table 1), according to the adjusted *P* value of 0.0083.

There was significant difference of iodine nutritional status between boys and girls (χ^2^ = 45.8, *P* < 0.01). The highest rate (26.9%) of UIC <100 μg/L was found to be those who provided non-iodized edible salt. More students had UIC ≥300 μg/L with inadequately or adequately iodized salt than those in the other two categories of salt-consuming groups (χ^2^ = 21.1, *P* < 0.01). Iodine nutritional status were not statistically different among age groups (χ^2^ = 5.1, *P* = 0.27, Table 2).

**Table 2:** Iodine status according to gender, age and category of table salt (*n*, %).

Characteristics	UIC (μg/L)			*P*
	<100	100–299	≥300	
Gender				<0.01
Boys	259 (9.3)	1667 (59.9)	858 (30.8)	
Girls	358 (12.9)	1767 (63.5)	656 (23.6)	
Age				
8 years	197 (11.5)	1073 (62.8)	439 (25.7)	0.27
9 years	203 (10.2)	1234 (62.0)	555 (27.9)	
10 years	217 (11.6)	1127 (60.5)	520 (27.9)	
Table salt				<0.01
Non-iodized	7 (26.9)	16 (61.5)	3 (11.5)	
Inadequately iodized	34 (9.4)	207 (57.3)	120 (33.2)	
Adequately iodized	560 (11.0)	3159 (62.0)	1375 (27.0)	
Excessively iodized	12 (18.5)	42 (64.6)	11 (16.9)	

Chi-squared test was used to compare the iodine nutritional status among gender, age and different salt-consuming subgroups. *P* < 0.05 was considered statistically significant.

### Thyroid size and goiter distribution

A proportion of 55.9% (3111) out of the 5565 students participated in the thyroid volume study. The TGR in all children was 1.9% (60/3111) and 6.0% (186/3111) according to Chinese national and WHO reference values, respectively. The goiter prevalence of children remained at a very low level according to Chinese domestic criteria among all subgroups, while more children were determined to be goitrous based on WHO criteria and the value indicated a status of mild IDD endemic. Thyroid size was non-normally distributed by KS test with D statistic of 0.08 (*P* < 0.01), and the median size was 2.9 mL (IQR: 2.5–3.4 mL). The thyroid volume and goiter rate among different subgroups were demonstrated in Table 3. Thyroid volume was found to be statistically different among age groups, which is normal according to natural growth law of adolescents. With WHO reference, goiter rates were statistically different among age groups with the highest value of 7.1% (69/971) in 8-year group, no statistical difference was identified among other subgroups.

**Table 3:** Thyroid size and goiter according to gender, age and category of table salt.

Characteristics	*n* (%)	Thyroid volume (mL)		Goiter rate^a^		Goiter rate^b^	
		Median (IQR)	*P*	*n* (%)	*P*	*n* (%)	*P*
Gender			0.12		0.43		0.44
Boys	1557 (50.1)	2.9 (2.5–3.4)		27 (1.7)		88 (5.7)	
Girls	1554 (50.0)	2.9 (2.5–3.4)		33 (2.1)		98 (6.3)	
Age			<0.01		0.34		<0.01
8 years	971 (31.2)	2.7 (2.3–3.1)		20 (2.1)		69 (7.1)	
9 years	1096 (35.2)	2.9 (2.5–3.4)		25 (2.3)		74 (6.8)	
10 years	1044 (33.6)	3.1 (2.7–3.6)		15 (1.4)		43 (4.1)	
Table salt			0.23		0.63^c^		0.93
Non-iodized	10 (0.3)	2.5 (1.9–3.1)		0 (0.0)		1 (10.0)	
Inadequately iodized	237 (7.6)	2.9 (2.5–3.3)		3 (1.3)		13 (5.5)	
Adequately iodized	2824 (90.8)	2.9 (2.5–3.4)		56 (2.0)		170 (6.0)	
Excessively iodized	40 (1.3)	3.1 (2.7–3.4)		1 (2.5)		2 (5.0)	

Wilcoxon test and Kruskal–Wallis test were used to compare thyroid volume among gender, age and different salt-consuming subgroups. Chi-squared test was used to compare goiter rate among subgroups. *P* < 0.05 was considered statistically significant.

^a^Calculated using domestic diagnostic criteria for endemic goiter (WS 276-2007). ^b^Calculated referencing gender-specific P97 according to WHO guide. ^c^Fisher’s exact test was used due to 38% of the cells had expected counts less than 5.

IQR, interquartile range.

### Correlation between iodine status and goiter by two references

**Figure 1:**
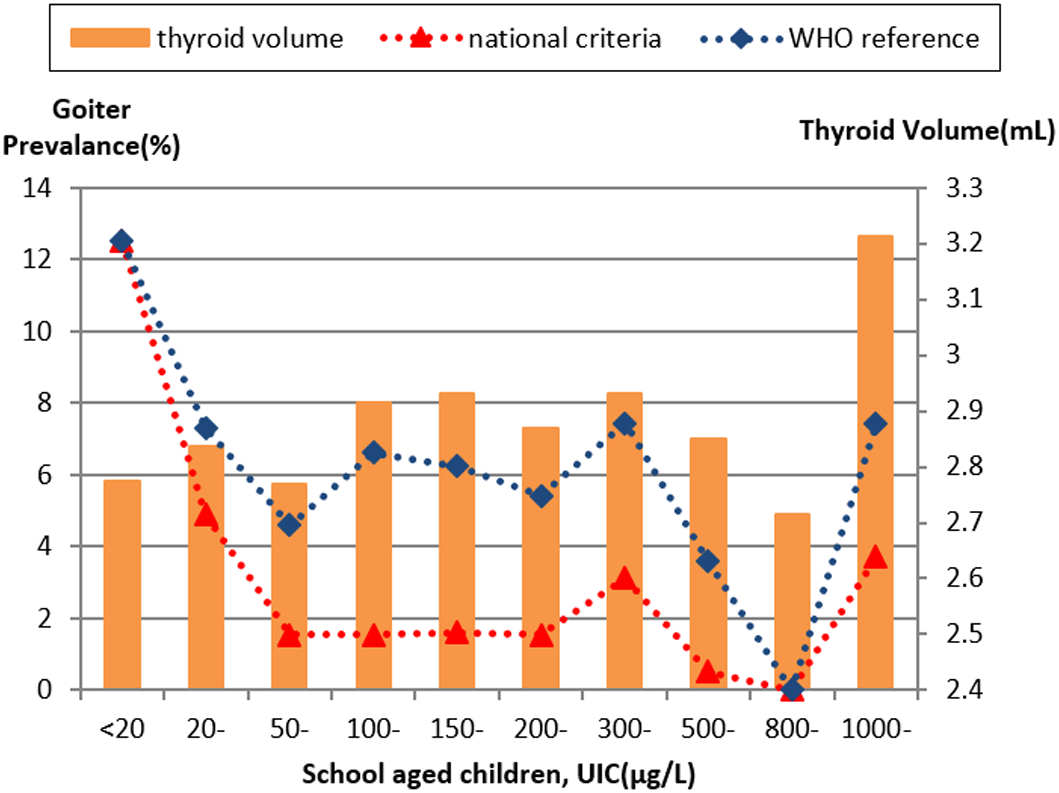
The thyroid volume and goiter prevalence in different UIC groups calculated using the WHO standard and the Chinese domestic criteria.

Goiter prevalence was not different within the three iodine nutritional status groups (*P* > 0.05, Table 4), but children has smaller goiter volume with UIC <100 μg/L (*P* = 0.04, Table 4). Multiple linear regression showed thyroid volume was correlated with age (*P* < 0.01), but not with UIC and content of table salt. The formulated curve described a slightly obvious ‘U shape’, which dropped quickly from UIC <20 μg/L group to 50–99 μg/L group, and then remained low and stable within the range of 50–500 μg/L groups. And dropped again when UIC >500 μg/L with the ends increased sharply as UIC >800 μg/L (Fig. 1). No association between thyroid volume and UIC was identified (*r* = −0.02, *P* = 0.25).

**Table 4:** Goiter prevalence and thyroid volume according to iodine status.

UIC (μg/L)	*n*	Goiter rate^a^	Goiter rate^b^	Thyroid volume (mL)
		*n* (%)	*n* (%)	Median (IQR)
<100	359	10 (2.8)	20 (5.6)	2.8 (2.4−3.3)
100-299	1919	30 (1.6)	114 (5.9)	2.9 (2.5−3.4)
≥300	833	20 (2.4)	52 (6.2)	2.9 (2.5−3.4)
*P*		0.15	0.90	0.04

^a^Goiter calculated using domestic diagnostic criteria for endemic goiter (WS 276-2007). ^b^Goiter calculated referencing gender-specific P97 according to WHO guide.

### Regional distribution of UIC and thyroid size

The range of median UIC from 26 areas was between 182 and 315 μg/L. A total of 25 districts/counties of Chongqing had median UIC between 100-299 μg/L, with one district with UIC over 300 μg/L. TGR of all the 14 areas that had received thyroid ultrasonic examinations for goiter were below 5% for Chinese national standard, ranging from 0.5 to 2.7%. Thyroid volume was between 2.6 and 3.1 mL. Eight (8) areas were assessed of iodine mild deficiency depending on TGR calculated by WHO criteria with a range of 5.0–10.5%.

## Discussions

Our results demonstrated the solid progress toward the implementation of USI program in Chongqing. Only 11.1% (617) of students have UIC <100 μg/L, and very few (5.2%) have UIC lower than 20 μg/L. A considerable proportion (61.7%) were assessed of having no iodine deficiency. Formal study had identified positive correlation between iodine content of drinking water and UIC, suggesting that high iodine content in drinking water was the main source of iodine excess for people living in those areas (1, 16, 17). Excess iodine in drinking water has also caused endemic goiter and thyroidal dysfunction in vulnerable populations within some areas of China (1, 18, 19). However, the environment in Chongqing is identified to be universally lacking iodine based on previous research involving water iodine investigation (2). The inappropriate use of iodized salt or excessive dietary iodine intake from the consumption of iodine-rich foods may have played some role in some children’s iodine excess in Chongqing.

Iodine concentration in household table salt has been changed three times based on the iodine nutritional status of population since it was initialized in 1993. At first, the national standard of edible salt was set at 40–60 mg/kg during 1993–1999, as the median UIC of children was found to be too high through national surveillance (5), it was changed to be 20–50 mg/kg in 2000. The last change of iodization level was reduced to 20, 25 or 30 mg/kg ± 30% in 2012, and each province has been mandated to choose its own iodine content, taking into account the actual iodine nutrition of the local population in order to avoid not only iodine deficiency but also iodine excess. Chongqing has chosen the highest level of 30 mg/kg (21–39 mg/kg). Our study showed the median UIC of students was 222 μg/L, consistent with the results of 2011 national IDD survey (5). UIC was identified to be statistically associated with iodine content of table salt, with the lowest UIC in the non-iodized salt group. The higher UIC was probably not explained by over-iodized salt as children with inadequately and adequately iodized salt had higher UIC than those with excessively iodized salt. Total amount of iodine intake daily may come from iodized salt, natural food that contain high iodine and processed food using iodized salt. Research has shown that iodized salt contributed 63.5% of food iodine, but 24.6% of this iodine is lost in cooking (20). As some students of the middle school might have lunch at school, accounting for iodine intake obtained from the edible salt used by the school kitchen is also important. While we did not collect dietary data for iodine intakes in the present study, more details of daily food intake and salt usage patterns are needed in order to further optimize the research for it is important that dietary sources of iodine and processed foods be identified and accounted for iodine intake in populations (21).

With the rapid progress in eliminating IDD around the world and the increased prevalence of thyroid disease, in particular goiter, thyroiditis, hyper-and hypo-thyroidism and thyroid cancer, risk of excessive iodine intake was gradually recognized. It is realized that iodine concentrations must be maintained at proper level as both deficiency and excess are harmful to thyroid (18). High goiter rate in children with high median UIC was observed in previous studies from areas in China with high iodine content in drinking water (1, 22). But when iodine is sufficient, children aged 6–12 years in Sweden showed no association between thyroid volume and UIC (23). Another international study of school students reported that median UIC are associated with increasing goiter rate when UIC >500 μg/L (24). No evidence of increased thyroid volume in school aged children with UIC lower than 300 μg/L was found (19, 25). Our study found no association between UIC and goiter prevalence, and the relatively stable and lower point of goiter rate of the ‘U shape’ curve located in the UIC range of 50–500 μg/L. It might be explained by two reasons given below. Firstly, the drinking water in Chongqing is universally lacking iodine and the median UIC of school aged children was not exceeding 500 μg/L. Our results indicated that children whose UIC were between 100 and 299 μg/L and children with UIC <100 μg/L or >300 μg/L both have the same goiter rate. Other studies also suggest that goiter and thyroid dysfunction seldom occur unless urinary iodine content exceeds 300 μg/L or higher (1, 24, 26). Our results further confirmed the hypothesis about the relationship between UIC and thyroid volume and agreed with the suggestion that the acceptable median UIC in children could be extended to a single category of adequate iodine nutrition of 100–299 μg/L (9, 26). Secondly, it is well known that goiter prevalence responds slowly to changes in iodine intake as thyroid size may not return to normal for month or years after correction of iodine deficiency (4). During this period, goiter reflects a population’s history of iodine nutrition instead of its present iodine status. The students whose UIC were in the optimal range of 100–299 μg/L and were assessed of iodine adequate in this survey might be goitrous before, with sustained slightly larger thyroids at this time. Although UIC may not provide direct and parallel relationship with thyroid size and function, the increased risk of developing thyroid disorders with both low and high UIC cannot be ignored (18). Excess dietary iodine has been associated with goiter and thyroid dysfunction in children and most of goiters could respond to restriction of dietary iodine intake (24). The children in our study who were already observed of having higher UIC and thyroid volume should reconsider the dietary iodine intake in daily life, as well as seek further medical care and be warned about potential risk for nodules, thyroiditis, hyper-and hypo-thyroidism and cancer development later in life.

In our study we observed discrepancies in goiter prevalence of school aged children between national ultrasound reference values with respect to those proposed by the WHO. The WHO reference was established by Zimmermann *et al*. (13), who proposed upper normative limits of thyroid volume values based on samples in long-term iodine sufficiency regions in six countries in Asian, Europe, Africa and America. The Chinese domestic criteria employed in this study were formulated in 2007 and was only age specific without adjusted for BSA and gender. Regional difference of endemic goiter prevalence do exist in China as there is a widespread distribution of areas with excessive iodine in drinking water in eleven provinces (22). It has been found that iodine excess caused by drinking water containing high iodine content and exacerbated by consumption of iodized salt has led to higher goiter prevalence (16). The Chinese criteria may be too high for areas with low iodine in drinking water like Chongqing and ignore gender differences and children’s variant body development of the same age. Goiter prevalence obtained in the study could be underestimated using these criteria. It is also hard to decide whether the WHO reference was appropriate for children’s goiter assessment in Chongqing as the results were lack of thyroid function and anti-thyroid antibody tests, which could be useful for thyroiditis diagnose and explain the reason of identified goiter (27). Further researches were needed to evaluate the applicability of WHO reference in goiter diagnose in Chongqing or identifying more accurate criteria of normal thyroid volume of local students in the future.

One limitation of the present study is that a random spot urine sample was collected for testing, which might be affected by variability in daily water intake (28) and intra-individual variations. The twenty-four-hour (24-h) urinary iodine excretion and creatinine (Cr)-standardized UIC are generally regarded as more reliable indicators to assess iodine nutrition. Secondly, we determined goiter rate based on both diagnostic criteria only adjusted for age without BSA which is also recommended by WHO (4). BSA requires the collection of children’s weights and heights in order to take account the differences in body development among children of the same age in different areas (12) and individual variations.

In conclusion, the results of our study confirmed that iodine prophylaxis, achieved by the USI program, is able to reduce the goiter prevalence as a low incidence of goiter in school aged children is still observed in Chongqing. Regular monitoring of progress toward sustaining elimination of IDD is essential in order to prevent both iodine deficiency and iodine excessive in children and pregnant women.

## Declaration of interest

The authors declare that there is no conflict of interest that could be perceived as prejudicing the impartiality of the research reported.

## Funding

This work was supported by the Chongqing Municipal Health and Family Planning Commission (grant number #2016MSXM100), the institution is now renamed as Chongqing municipal Health Commission.

## Author contribution statement

Z S conceptualized the study design. X J and L XS performed project administration and supervision. Z CB and Y N performed statistical analyses, data interpretation and wrote the manuscript. Z QR and C J provided laboratory support and completed the quality control work. Z S critically revised the manuscript and approved the final version to be submitted. All authors read and approved the final manuscript.
